# Probing the Dynamic Process of Encapsulation in *Escherichia coli* GroEL

**DOI:** 10.1371/journal.pone.0078135

**Published:** 2013-10-30

**Authors:** Toshifumi Mizuta, Kasumi Ando, Tatsuya Uemura, Yasushi Kawata, Tomohiro Mizobata

**Affiliations:** 1 Department of Biotechnology, Graduate School of Engineering, Tottori, Japan; 2 Department of Biomedical Science, Graduate School of Medical Sciences, Tottori University, Tottori, Japan; Duke University Medical Center, United States of America

## Abstract

Kinetic analyses of GroE-assisted folding provide a dynamic sequence of molecular events that underlie chaperonin function. We used stopped-flow analysis of various fluorescent GroEL mutants to obtain details regarding the sequence of events that transpire immediately after ATP binding to GroEL and GroEL with prebound unfolded proteins. Characterization of GroEL CP86, a circularly permuted GroEL with the polypeptide ends relocated to the vicinity of the ATP binding site, showed that GroES binding and protection of unfolded protein from solution is achieved surprisingly early in the functional cycle, and in spite of greatly reduced apical domain movement. Analysis of fluorescent GroEL SR-1 and GroEL D398A variants suggested that among other factors, the presence of two GroEL rings and a specific conformational rearrangement of Helix M in GroEL contribute significantly to the rapid release of unfolded protein from the GroEL apical domain.

## Introduction

Analyses of macromolecular complexes such as the *E. coli* chaperonin GroE involve a multitude of functionally relevant events, dynamic transitions of the molecular machinery, and various structural characteristics of the molecular architecture that must all be united coherently and consistently in order to understand the underlying molecular mechanism in detail. To date, many studies have succeeded in elucidating and correlating these varied details of the chaperonin to form a well defined overview of the molecular mechanism, and recent experiments are involved in forming increasingly detailed views of various segments of the mechanism [1]–[3]. One such segment involves the process by which GroEL transfers proteins into the protective central cavity formed by the unique double ringed quaternary structure of the 14 GroEL subunits [4]–[7]. This process is initiated by binding of ATP to GroEL and involves intermolecular interactions between the unfolded protein molecule and the co-chaperonin, GroES.

Various studies have indicated that the process of protein encapsulation initiated by ATP binding to GroEL is a multi-step transition, involving many experimentally distinguishable substates that must be formed to complete the final, sequestered state of the chaperonin complex [8]–[11]. Cryo-electron microscopy (cryo-EM) experiments have revealed a number of snapshots of individual conformations that GroE forms during this process [12], [13]. In stopped-flow experiments, a number of groups including ours have shown that this process involves numerous distinct structural transitions with varying kinetic characteristics [14]–[21]. The challenge at present is to meld these structural snapshots (*e. g.*, electron microscopy) and kinetic (*e. g.* stopped-flow) details of the chaperonin mechanism with functional aspects to form a unified mechanism of the overall process. Clare and coworkers have recently identified numerous substates of *E. coli* GroEL D398A that are formed immediately after ATP binding [13]. From a principle of minimum structural alterations between substates, they postulated a sequential mechanism that describes the initial molecular trajectory of GroE encapsulation that culminates in GroES binding and formation of the capsule.

**Figure 1:**
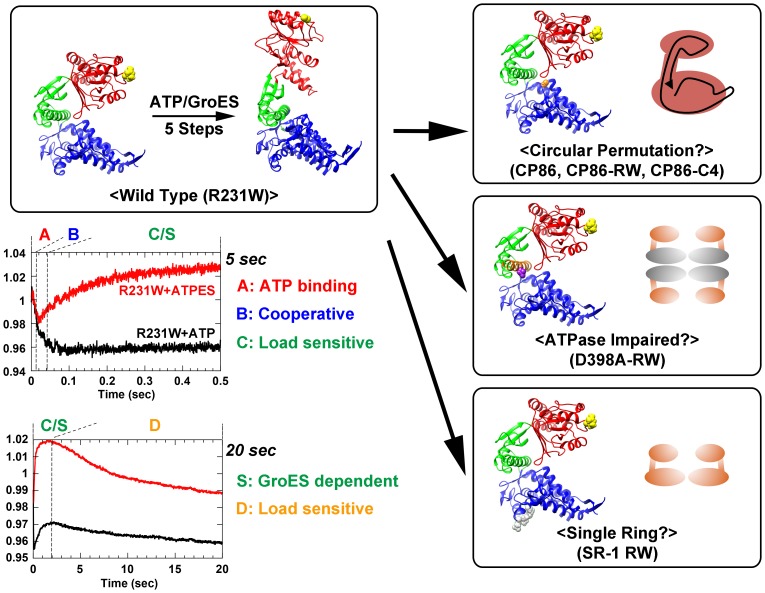
Conceptual diagram and subunit structures depicting the various mutations used in the present study. GroEL R231W is a fluorescent mutant of GroEL with wild-type like functional characteristics (*upper left*). Stopped-flow analysis using this mutant revealed a total of five distinct kinetic transitions that are triggered immediately after ATP binding (denoted Phases A∼D and Phase S; *lower left*), and each phase displays characteristics relevant to the chaperonin mechanism (experimental traces are derived from previous experiments described in Yoshimi *et al* [20]). In the present study, this mutation was introduced to the corresponding sites of three separate mutants: CP86, a circularly permuted GroEL mutant (*upper right*; Gly86 is shown in *orange space filled form*), D398A, a mutant with greatly diminished ATPase activity (*center right*; Asp398 is shown in *purple space filled form* and Helix M is colored *orange*), and SR-1, a single ring GroEL variant (*lower right*; the four residues of the SR-1 mutation, Arg452, Glu461, Ser463, and Val464, are shown in *gray space filed form*). Stopped-flow analysis was performed to evaluate the effect of each mutation on the dynamics of GroEL reported by this tryptophan probe. In each of the representative subunit structures, Arg231 is shown in *yellow space filled form*, and the three domains of the GroEL subunit are colored as follows: apical; *red*, intermediate; *green*, equatorial; *blue*. Models were visualized in UCSF Chimera [41].

The present study represents our attempts to find kinetic evidence that may add to our understanding of the chaperonin encapsulation mechanism, utilizing a mutation that introduces a unique tryptophan into the GroEL apical domain (GroEL R231W, Fig. 1, *top left*). Using the original GroEL R231W mutant, we have demonstrated that GroEL undergoes five kinetically distinguishable transitions (Phases A to D, Phase S) during the process of encapsulation (Fig. 1, *lower left*; [20]). Each of these kinetic transitions displays characteristics that are relevant to the chaperonin mechanism. For example, the apparent rate constant of one specific kinetic transition (Phase B, fluorescence decrease, *k*_max_ = 82 s^−1^) displays complex cooperative behavior with regard to ATP concentration [21], and is postulated to represent intersubunit rearrangements. Also, another phase (Phase C, fluorescence increase, *k*_max_ = 2 s^−1^) likely reflects a localized movement that involves displacement of the unfolded protein bound to the apical domain of the initial binary complex, because the rate of this specific phase is sensitive to bound substrate protein [20]. Phase D is also sensitive to the presence of bound unfolded protein (being suppressed upon binding of unfolded MDH to GroEL, [20]), and Phase S is observable only in the presence of the co-chaperonin GroES [20] (Fig. 1).

Building upon these prior findings, we describe here our efforts to elucidate the molecular characteristics of GroEL function using various fluorescent mutants tailored for stopped-flow analysis (Fig. 1). Notably, we include our findings using a newly characterized circular permutation GroEL mutant, CP86 (Fig. 1, *top right*). CP86 is a variant of the GroEL subunit where the polypeptide termini have been relocated to the immediate vicinity of the ATPase site. Experiments showed that this mutant displayed almost none of the kinetic transitions shown by the wild type chaperonin. Despite this radical alteration, we determined that this mutant was capable of binding GroES and protecting unfolded protein molecules from extraneous protease digestion, a characteristic previously attributed to the successful completion of protein encapsulation. Our results suggest that the encapsulation of unfolded protein molecules into the GroEL chamber may proceed through two sequential processes, a preliminary process involving GroES binding that accords the unfolded protein a significant amount of protection from the environment, followed by a subsequent large scale rearrangement of the GroEL subunit and the apical domain. We also show results obtained from fluorescent variants of the ATPase-impaired mutant D398A (Fig. 1, *center right*) [10]–[12] and the single-ring GroEL mutant, SR-1 (Fig. 1, *bottom right*) [8], [9] that suggest that encapsulation of unfolded proteins by GroE is essentially a multi-stage sequential process whose smooth execution is dependent on numerous structural factors of the GroEL architecture.

## Materials and Methods

### Proteins and Purification

GroEL CP86 was a circularly permuted mutant isolated from an initial screening of randomly permuted GroEL genes described in Mizobata *et al* [22]. The mutant was selected for its relatively stable expression in the supernatant fraction of *E. coli* cells. As described in Mizobata *et al.*, circularly permutated GroEL variants obtained *via* our protocol contain several extraneous amino acids that are not found in the original GroEL amino acid sequence.

Two derivatives of the CP86 mutant were created in addition for use in further experiments. The CP86-C4 mutant was constructed in order to determine the specificity and reversibility of the circular permutation effect, and this mutant possesses four cysteine residues (Cys3, Cys4, Cys552, Cys553) dispersed at both ends of the polypeptide chain. The CP86-RW mutant is a derivative of CP86 with a tryptophan residue in the apical domain, in the position corresponding to Arg231 in the wild type sequence (position 149 in the actual mutant sequence), and this mutant was used in stopped-flow studies. Construction of the CP86-C4 and CP86-RW mutants were performed using the Quikchange site directed mutagenesis kit (Agilent Technologies) for point mutations. Additionally for CP86-RW, the terminal amino acid sequences were modified slightly in an attempt to improve expression. Details of the amino acid sequences are summarized in Table 1. The nucleotide sequences of the CP86, CP86-RW and CP86-C4 GroEL genes have been deposited in the GenBank database under GenBank Accession Number KC355805 (CP86), GenBank Accession Number KC415072 (CP86-RW), and GenBank Accession Number KC355806 (CP86-C4).

**Table 1:** N- and C-terminal sequences of circularly permuted GroEL mutants CP86, CP86-RW, and CP86-C4.

Mutant	C-terminal sequence	N-terminal sequence
CP86	ANDAAG*AN* ^555^	^1^ *MAA*GDGTT
CP86-RW	ANDAA^550^	^1^ *M*GDGTT
CP86-C4	ANDA **CC** *AN* ^555^	^1^ *MA* **CC**DGTT
*Corresponding* *wild type sequence*	^81^ ANDAAGDGTT^90^	

Underlines and wavy underlines denote the corresponding amino acid sequences in the original wild type sequence. In GroEL CP86-RW, an additional Arg to Trp mutation was introduced at position 149 in the mutant sequence. Amino acids in *italics* are residues that were introduced as a consequence of the circular permutation protocol, the four cysteine residues introduced to the CP86-C4 sequence are **bolded**.

The construction and characterization of GroEL SR-1 RW has been previously published in Taniguchi *et al* [21]. GroEL D398A-RW was constructed using Quikchange site directed mutagenesis. Purification of GroEL SR-1 RW and D398A-RW were performed as described previously for GroEL SR-1 RW [21]. Both of these mutants were purified to sufficient purity for stopped-flow studies. Characterization of D398A-RW showed that, as in the original D398A mutant, the ATPase activity of this mutant was reduced greatly compared to the wild type. Purification of CP86 GroEL and derivatives was accomplished according to methods used to purify wild type GroEL [23]. However, after the final stage of the purification, an additional acetone precipitation step was added to enhance the purity of the samples. Ice-cold acetone was added to a final concentration of 40% v/v to GroEL samples (5 mg/ml) incubated on ice with mixing. After a 30 min incubation on ice, samples were centrifuged at 15,000×g for 30 min and the supernatant was recovered and dialyzed at 4°C against 50 mM Tris-HCl, pH 7.8 containing 2 mM EDTA overnight.

### Preparation of Oxidized CP86-C4 Samples

Samples of GroEL CP86-C4 were mixed with 2 mM ATP in oxidizing buffer (50 mM Tris-HCl, pH 7.8, containing 10 mM magnesium acetate, 20 mM KCl, 1 mM EDTA, 5 mM oxidized glutathione, and 1 mM reduced glutathione) and then incubated at 37°C for 1 hr. After incubation, samples were dialyzed against dialysis buffer (50 mM Tris-HCl, pH 7.8 and 2 mM EDTA), quantitated, and used in various assays.

### ATPase Assay

ATPase assays of GroEL CP86 and derivatives were performed colorimetrically as described in Kubo *et al* [24].

### Refolding Assays

Refolding assays of bovine rhodanese and pig heart malate dehydrogenase (MDH) were performed according to previously published procedures [22], [25]. Samples of MDH were obtained commercially from Roche Diagnostics; recombinant rhodanese expressed in *E. coli* was purified according to the purification protocol described in Miller *et al* [26]. The expression vector for rhodanese that we used contains an *E. coli* codon-optimized variant of the bovine rhodanese cDNA sequence, synthesized by Gene-Art (Germany).

### Proteinase K Protection Assays of Unfolded Proteins Bound to Chaperonin

Proteinase K protection assays of beryllium fluoride (BeFx)-stabilized chaperonin:substrate complexes were performed as described in Mizobata *et al* [22].

### Electron Microscopy

Electron microscopy of CP86 samples negatively-stained with uranyl acetate were performed essentially as described in Mizobata *et al*. [22], using a JEOL 1210 electron microscope at 80 kV.

### Stopped-flow Fluorescence Analysis

Stopped-flow fluorescence analysis of GroEL mutants was performed as described in Mizobata *et al.* [22], using an upgraded Applied Photophysics SX17MV instrument at a photomultiplier voltage of 400 V. The oligomer concentration of GroEL during measurement was set to 0.625 µM for all experiments, and equimolar oligomeric concentrations of GroES were added to the nucleotide-containing reagents where indicated. The concentration of nucleotide added was set to 1 mM during measurement in the present study. In experiments that probed the effects of prebound MDH, we first denatured MDH samples (5 mg/ml) for 30 min at 37°C in 3 M guanidinium hydrochloride (Gdn-HCl) containing 6.25 mM dithiothreitol. This denatured MDH sample was then added to aliquots of GroEL (1 mg/ml) so that the ratio of MDH monomer to GroEL oligomer was 1∶1 in SR-1 RW samples and 1∶2 in GroEL R231W samples. The higher concentration of MDH concentration in the R231W samples was used in order to achieve binding of MDH to both rings of the chaperonin. These chaperonin-MDH samples were used after a 5 min pre-incubation at 25°C in stopped-flow experiments.

Experiments involving FRET between the tryptophan residue of GroEL mutants and chemically-labeled unfolded GroEL wild type (GroELwt) have been previously described in Yoshimi *et al* [20]. Briefly, 20 mg/ml GroELwt was incubated with 5 mM 5-((((2-Iodoacetyl)amino)ethyl)amino)naphthalene-1-sulfonic acid (IAEDANS) in 50 mM Tris-HCl buffer, pH 7.5, at 25°C for 30 min. After incubation, 10 mM 2-mercaptoethanol was added to stop the reaction and the sample was dialyzed overnight against 50 mM Tris-HCl buffer, pH 7.5 containing 0.5 g/l acid-washed charcoal. The ratio of incorporated label to GroELwt subunit in samples obtained by this procedure was generally between 0.2 to 0.5 (quantitated using the molar extinction coefficient of 5,700 cm^−1^M^−1^ for AEDANS at 336 nm). AEDANS-GroELwt was denatured in 20 mM glycine-HCl buffer, pH 2.0, for 1h at 25°C and then mixed with an equimolar concentration of GroEL mutants. Data were first obtained using GroEL mutants with prebound AEDANS-GroELwt, and then data obtained in the absence of AEDANS-GroELwt was subtracted from this trace to obtain the FRET traces.

To facilitate comparison, raw fluorescence intensity traces in each figure have been shifted vertically so that the fluorescence value for each trace at the earliest data point equals 1. Fits to the raw data are displayed as white lines running through the trace in each panel; residuals are summarized in a sub panel below the main panel of each figure. Amplitudes and apparent rate constants derived from the fits are summarized in Table S1 (tryptophan fluorescence) and Table 2 (FRET).

**Table 2:** Values of the kinetic constants derived from analyses of FRET traces shown in Fig. 8.

	PhaseFRET		PhaseFRET2	
	Amplitude	*k*(sec^−1^)	Amplitude	*k*(sec^−1^)
GroEL SR-1	0.0051±0.00021	4.1±0.29	0.0024±0.000067	0.21±0.012
GroELD398A-RW	0.002±0.00024	1.8±0.46	0.0016±0.00015	0.18±0.034

## Results

In the present study, we introduced the GroEL R231W mutation [21] into the corresponding site of three unrelated GroEL mutants (Fig. 1). The results obtained from each mutant addresses a specific section of the encapsulation mechanism that, when combined, provide details regarding the rapid events that follow immediately after ATP binding, culminating in the formation of an encapsulation complex containing GroEL, GroES, and unfolded protein. The stopped-flow fluorescence results are interpreted in the context of the results of GroEL R231W, which represents the kinetic behavior of wild type chaperonin.

### GroEL CP86: Protein Sequestering and Subunit Dynamics

We have recently incorporated circular permutation in our research as a method to perturb the GroEL subunit and to induce changes in its function. Circular permutation involves modifying the amino acid sequence of the target protein so that N-terminal and C-terminal amino acids are shifted to a different portion of the protein molecule [27]. Application of random circular permutation to the *E. coli* GroEL subunit [22] produced a mutant (GroEL CP376) that was used in a previous study to demonstrate that Phase C exclusively reflects localized movements of the GroEL apical domain.

**Figure 2:**
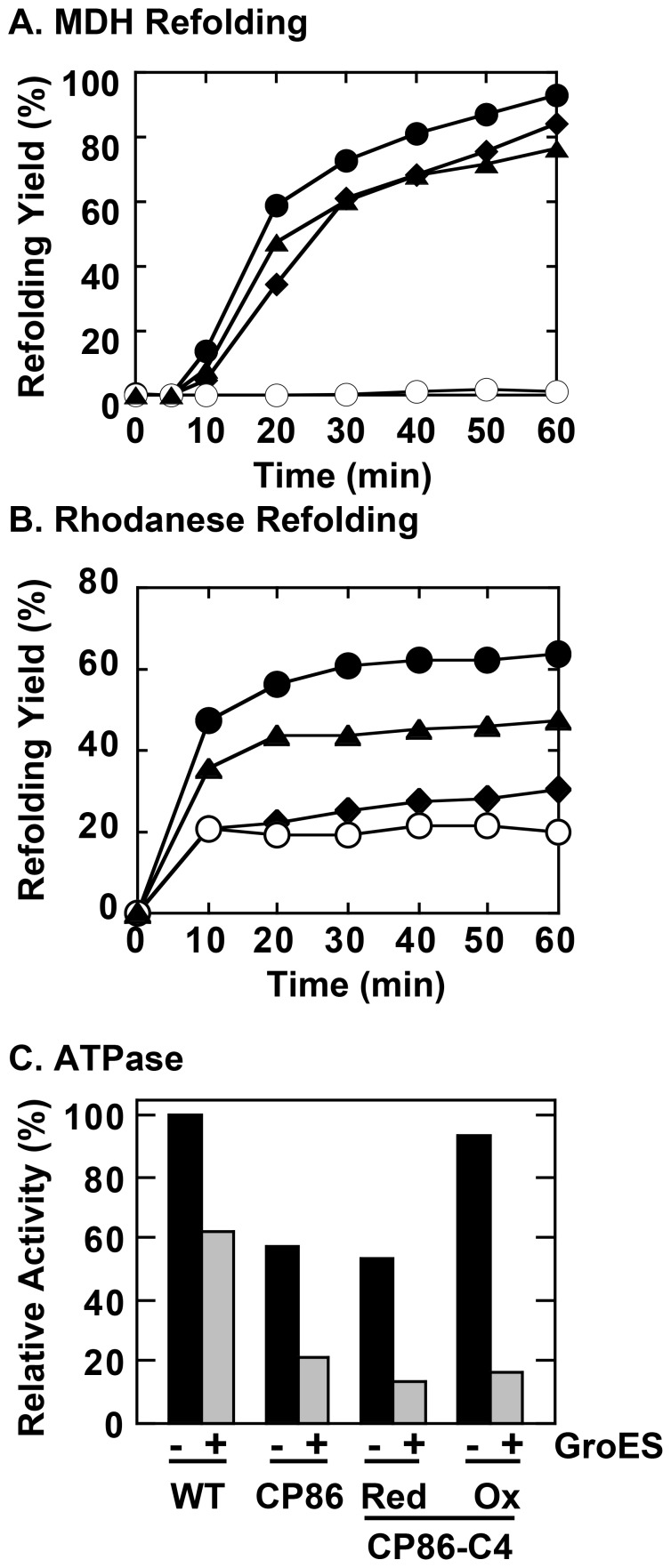
Functional assays of GroEL CP86 and oxidized glutathione-treated GroEL CP86-C4. A. Refolding assays of pig heart MDH. *Closed circles* denote refolding in the presence of wild type GroEL and GroES, *open circles* denote spontaneous refolding. *Closed diamonds* indicate refolding in the presence of CP86 and wild type GroES, and *closed triangles* indicate refolding in the presence of CP86-C4 pre-treated with oxidized glutathione in the presence of ATP. B. Refolding assays of bovine rhodanese. Legends are identical to panel A. C. ATPase activity of GroEL mutants. The concentration of inorganic phosphate released after 60 min is given as values relative to the basal ATPase activity seen for wild type GroEL in the absence of equimolar GroES (*leftmost solid bar*).

GroEL CP86 was a circularly permuted variant of GroEL that was also isolated in this initial effort, whose polypeptide ends were relocated to a position between residues 85 and 86, in the immediate vicinity of the ATP binding site in the equatorial domain (Fig. 1 and Table 1). Figs. 2A and B show refolding assays of MDH and rhodanese in the presence of GroEL CP86. The refolding assays showed that CP86 displayed differential effects toward the stringent refolding substrates rhodanese and MDH. The effects were very clear-cut; the ability to facilitate folding of MDH was completely retained in CP86 (Fig. 2A, *closed diamonds*) whereas the ability to assist rhodanese was decreased almost to insignificance (Fig. 2B, *closed diamonds*). In addition to this striking characteristic, the functional activity of CP86 was characterized by a decreased basal ATPase activity that remained sensitive to inhibition by GroES (Fig. 2C).

Our next step before proceeding further was to evaluate the specificity of the effects caused by the circular permutation. Since circular permutation in effect causes the disruption of the polypeptide backbone, we reasoned that it might be possible to reverse these effects by inserting a disulfide bond to the mutation site. To this end we engineered another derivative of CP86 (CP86-C4), which contains tandem cysteine residues at both ends of the polypeptide chain (Table 1).

**Figure 3:**
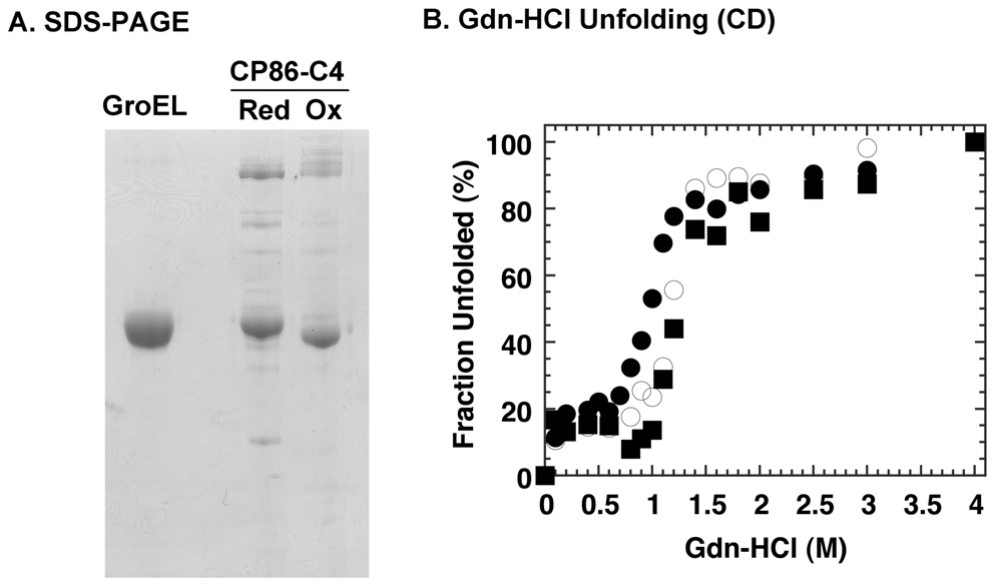
Characterization of CP86-C4 samples treated with oxidized glutathione. CP86-C4 was treated with oxidized glutathione as outlined in Materials and Methods. A. SDS-PAGE analysis of CP86-C4 samples in the absence of 2-mercaptoethanol, which shows a shift in gel position caused by formation of an intramolecular disulfide bond. B. Gdn-HCl unfolding profiles of wild type GroEL (*open circles*), GroEL CP86 (*closed circles*), and oxidized GroEL CP86-C4 (*closed squares*) monitored by the CD signal at 222 nm. GroEL was incubated in HEPES-KOH buffer, pH 7.8, containing 10 mM KCl, 5 mM magnesium acetate, and the indicated concentration of Gdn-HCl for 2 h at 25°C prior to measurement. A JASCO J-820 spectropolarimeter was used to measure the CD signal.

When we incubated CP86-C4 with oxidized glutathione in the presence of ATP, we induced functional and structural alterations to this mutant. Figs. 3A and B show experiments that demonstrate the effects of this treatment on the structural characteristics of CP86-C4, namely a shift in the gel mobility in non-reducing denaturing electrophoresis gels and an increased stability toward denaturation by Gdn-HCl. Both of these results support the formation of a disulfide bond between the polypeptide termini of CP86-C4 via the introduced cysteine residues.

Upon repeating the experiments performed on the original CP86 mutant, we now found that the oxidized CP86-C4 recovered its ability to assist the folding of rhodanese (Fig. 2B, *closed triangles*). Additionally, ATPase activity assays showed that the basal ATPase activity also increased to levels similar to the wild type chaperonin (Fig. 2C).

**Figure 4:**
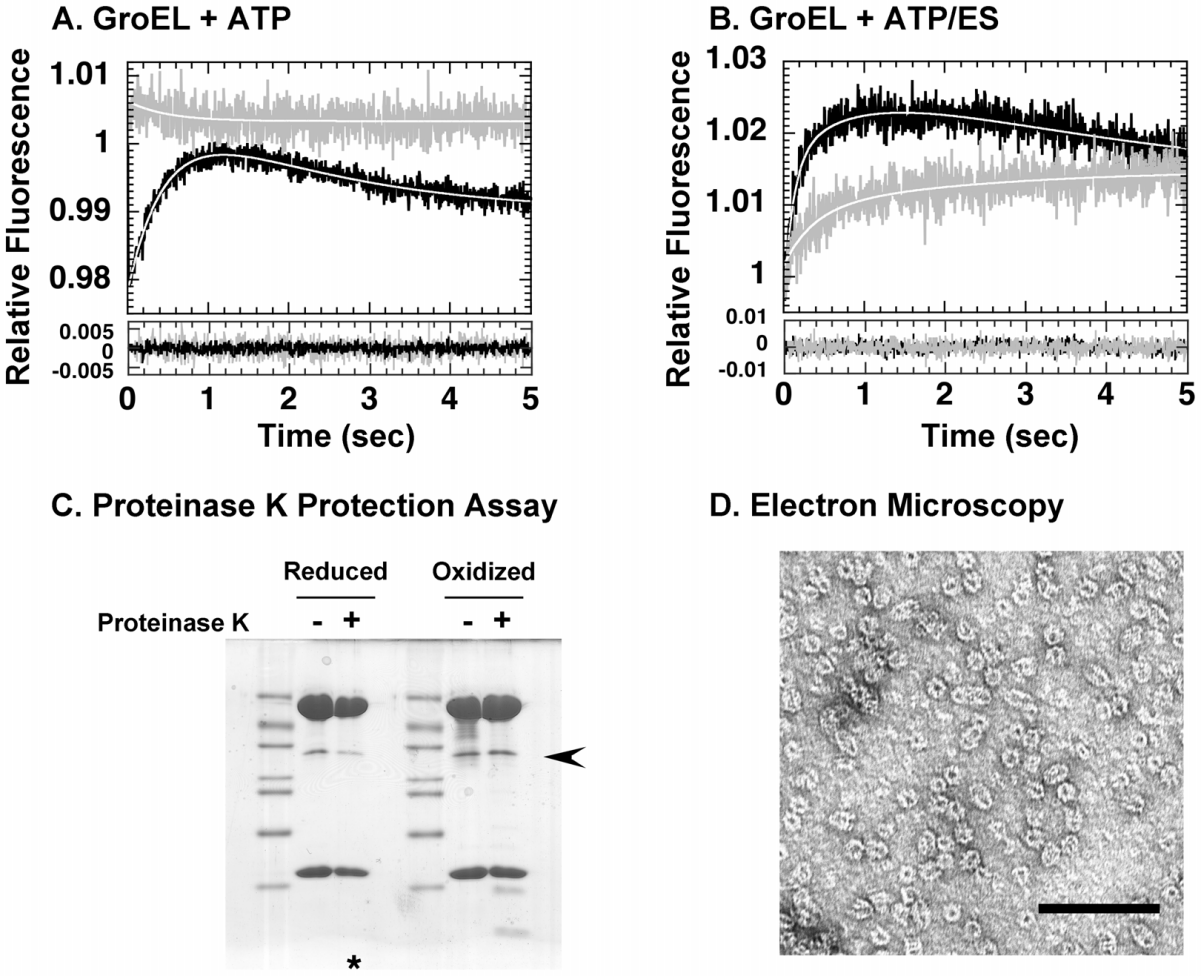
Analysis of the chaperonin cycle using CP86 variants. A. Stopped-flow analysis of GroEL CP86-RW upon addition of 1 mM ATP. *Black traces* correspond to GroEL R231W and denote wild-type like behavior. *Gray traces* correspond to GroEL CP86-RW. In this and all subsequent figures that involve fits of raw data to theoretical curves, the *lower panel* summarizes the residuals derived from each fit, with legends corresponding to the upper panel. B. Stopped-flow analysis of GroEL CP86-RW upon addition of ATP and equimolar GroES. Legends are as in A. C. Proteinase K protection assays of rhodanese molecule bound to oxidized and reduced GroEL CP86-C4. Samples shown are from solutions concentrated on an Amicon Ultra-0.5 100K centrifugal filter unit after a 30 min digestion with 1 µg/ml Proteinase K at 25°C. The *arrowhead* denotes the position of rhodanese. The asterisk at the bottom of the gel indicates the sample that was subsequently used to prepare negatively stained samples analyzed in panel D. D. Electron micrographs of BeFx-stabilized, Proteinase K-treated samples of GroE-rhodanese ternary complexes prepared using reduced GroEL CP86-C4. Scale bar denotes 100 nm.

The refolding reactions of MDH and rhodanese most likely contained previously undetected differences that become apparent when folding in the presence of CP86. In order to probe this interesting finding in more detail, we next probed the dynamics of this mutant by stopped-flow analysis using a fluorescent analog of CP86, CP86-RW. Functional characteristics of the original CP86 mutant were retained in this new fluorescent variant. As shown in Fig. 4A, we found that in this mutant, ATP-triggered kinetic transitions involving the apical domain were greatly decreased, and consisted of an initial increase in fluorescence reminiscent of Phase A, followed by an extremely slow decrease in fluorescence, which might represent a greatly attenuated Phase B. Upon addition of GroES to the experiment (Fig. 4B), an increase in fluorescence was observed that could be fitted to a double exponential equation to yield two kinetic phases reminiscent of Phases C and S in GroEL R231W. The values of the rate constant and amplitude, when compared to the putatively corresponding phases in GroEL R231W, were both significantly attenuated, however (Table S1).

Next, we evaluated the ability of CP86 to encapsulate and protect unfolded rhodanese, by adding ATP, GroES, and BeFx to GroEL:rhodanese binary complexes and digesting the stabilized sample with Proteinase K. To our surprise, we found that GroEL CP86-C4 in its reduced state was capable of protecting unfolded rhodanese molecules even though it could not assist folding (Fig. 4C). Electron microscopy of these Proteinase K-treated chaperonin samples clearly showed CP86-C4 forming both typical bullet-like and football-like structures (Fig. 4D). Taken together with the stopped-flow results we conclude that CP86 is capable of protein encapsulation and protection in a manner similar to wild type GroEL, even when dynamic changes involving the apical domain are greatly restricted by mutation.

### GroEL D398A: Domain Movement and ATP Hydrolysis

To obtain hints regarding the relationship between the kinetic transitions detected by stopped-flow analysis and the ATPase activity of GroEL, we next utilized the unique characteristics of GroEL D398A, which displays an ATPase activity rate that is ∼2% of wild-type [10], [11].

**Figure 5:**
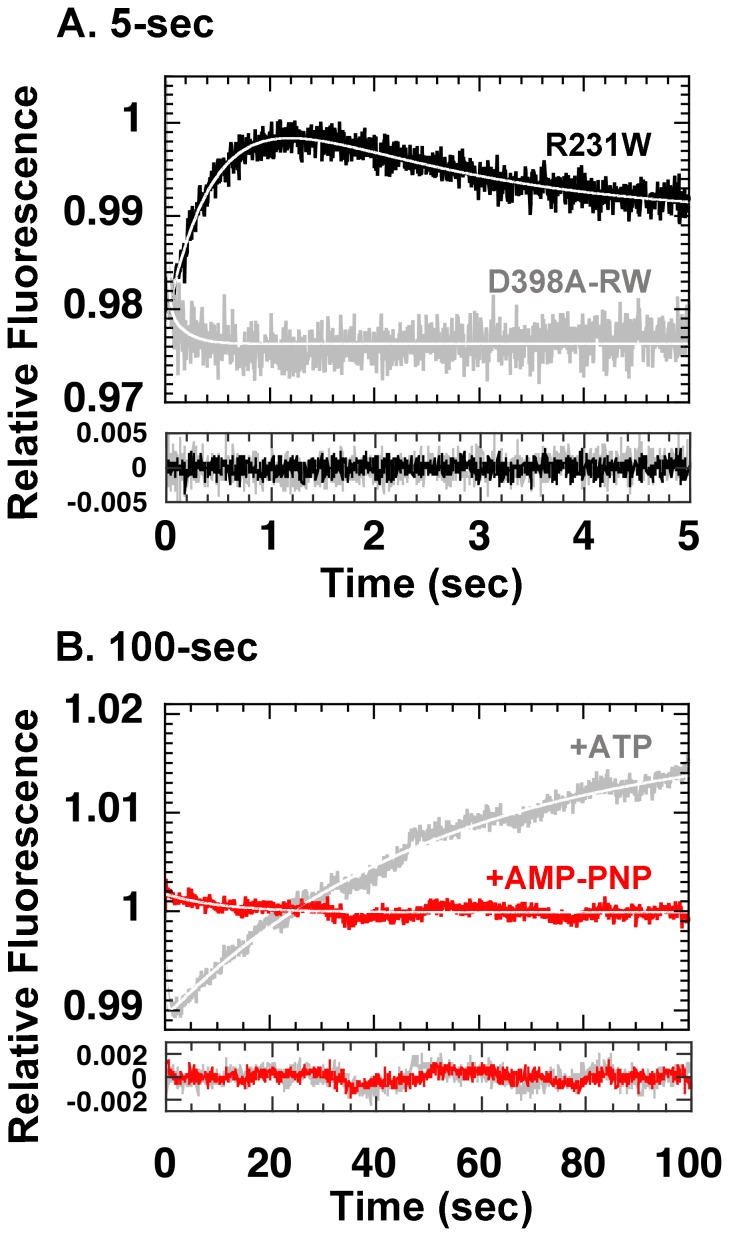
Stopped-flow analysis of GroEL D398A-RW. A. Analysis of fluorescence intensity changes upon addition of 1*black trace* indicates GroEL R231W, and the *gray trace* indicates D398A-RW. B. As in A, but with the duration of observation set to 100 sec. *Gray trace* indicates D398A-RW with ATP added (as in A), *red trace* indicates fluorescence changes upon addition of 1 mM AMP-PNP.

As seen in Fig. 5A, we found that in GroEL D398A-RW, the kinetic profile observed upon ATP addition was altered, in a manner similar to that seen for CP376 characterized previously [22]. The effects of mutation were seen most strongly in Phase C, while other transitions such as Phase B were relatively untouched. When we extended our observations to ∼100 sec after ATP addition however, we found that Phase C was not in fact suppressed in GroEL D398A-RW, but rather the rate was greatly decreased (Fig. 5B). Analysis of the apparent rate constant of this phase yielded a value that was decreased more than sixty-fold (*k* = 0.019±0.00029 s^−1^) compared to the wild-type analog (*k* = 2.1±0.11 s^−1^ for GroEL R231W; Fig. 4A, Table S1).

**Figure 6:**
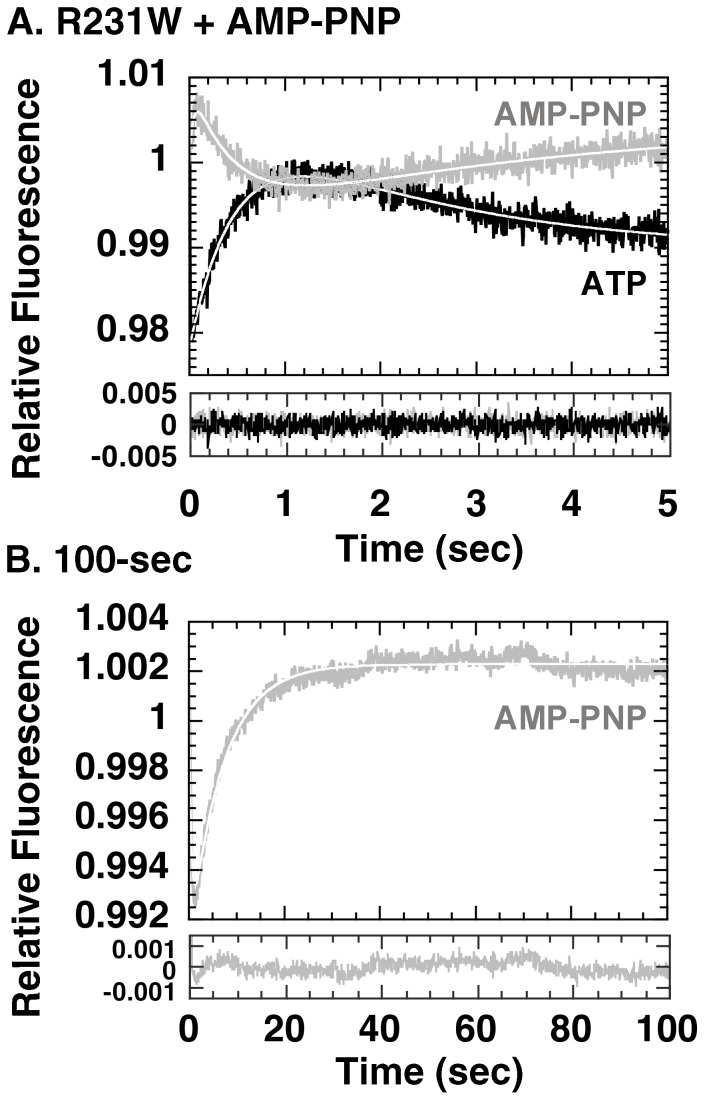
Stopped-flow analysis of GroEL R231W in the presence of AMP-PNP. A. 5-sec assay. The *black trace* represents fluorescence changes upon addition of ATP, the *gray trace* represents fluorescence changes upon addition of AMP-PNP. B. 100-sec assay. Legends are as in A.

Since we found it revealing that such a drastic decrease in rate could be observed in the ATPase-limited D398A mutant, we probed further by performing similar stopped-flow experiments using non-hydrolyzable AMP-PNP. We found that in the presence of AMP-PNP, Phase C was now completely suppressed for the duration of the experiment (Fig. 5B). A very gradual and ill-defined fluorescence decrease was detected instead, after an initial small fluorescence increase. From Fig. 5B it seemed possible a dependency might exist between Phase C and scission of the **β**-**γ**-phosphoanhydride bond of ATP *i. e.,* Phase C may be triggered only after bond scission. If this were true, however, this sequence of events should also apply to the wild type chaperonin. Therefore, to clarify this point we performed the same experiment as shown in Fig. 5B, but now using GroEL R231W, which displays wild-type behavior (Fig. 6). Interestingly, in the presence of AMP-PNP, Phase C could be clearly detected in GroEL R231W, although with a greatly decreased rate (Fig. 6A and B). Binding of the non-hydrolysable analog seemed to result in a decreased rate of fluorescence change for all of the phases detected in the experiment (*k = *25±4.4 s^−1^, 2.2±0.22 s^−1^, 0.29±0.084 s^−1^ for Phases A, B, and C, respectively, compare with corresponding values for *k* in [21], Fig. 6A and Table S1), and we could also detect a decrease in the amplitude for Phase B as well. The amplitude of Phase C, in contrast, seemed to be affected only minimally by the substitution of ATP by AMP-PNP. These results argued against a dependent relationship between ATP hydrolysis and the initiation of Phase C.

### GroEL SR-1; Substrate Protein Detachment and GroEL Quaternary Structure

**Figure 7:**
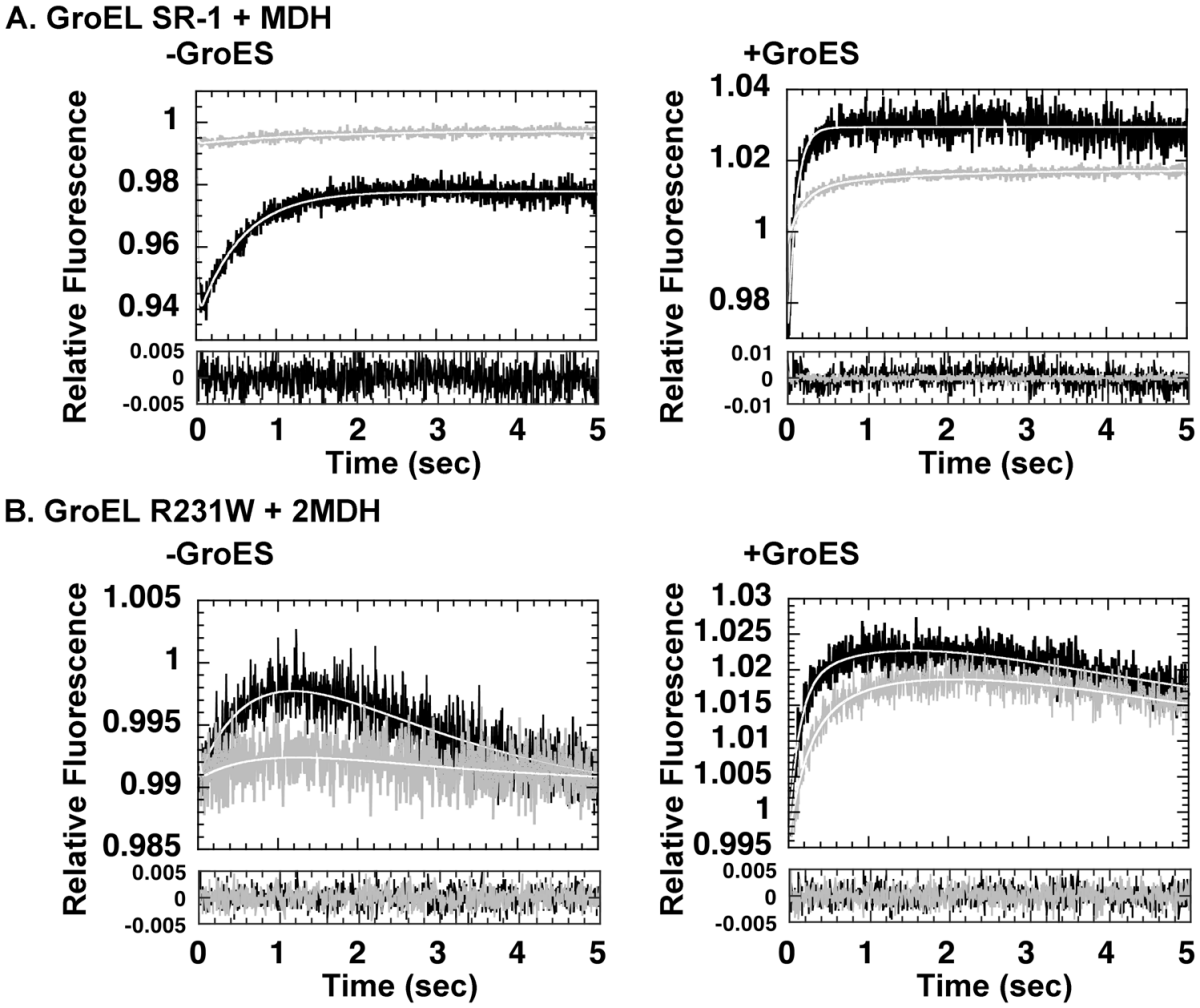
Stopped-flow analysis of SR-1 RW and GroEL R231W: dependence on prebound MDH and GroES. A. (*upper panels*) Analysis of SR-1-RW initiated by addition of ATP only (*left*, “-GroES”) or ATP and equimolar GroES (*right*, “+GroES”). *Black traces* indicate GroEL SR-1 RW only, *gray traces* indicate SR-1 RW with prebound MDH. B. (*lower panels*) As in A., but performed using GroEL R231W instead of SR-1 RW in each experiment.

The third GroEL mutant that we chose for our experiments was the single ring mutant SR-1, frequently used in many studies to analyze the GroE mechanism. We have already characterized the fluorescent derivative of SR-1 (SR-1 RW) to a certain extent in Taniguchi *et al.* [21], and we confirmed that the basic kinetic characteristics of SR-1 RW were similar to that of the original R231W mutant, with the notable exception that in the absence of GroES, Phase D, the slow fluorescence decrease seen in the final segment of the kinetic trace, was missing in SR-1 RW (compare the *black traces* in the *left-side panels* of Fig. 7A and 7B). This last result was a new finding that suggested a dependence of this kinetic phase to the characteristic double ring structure of GroEL. Here we probed the effects of a prebound substrate protein, Gdn-HCl unfolded MDH, on the kinetic profile of SR-1 RW (Fig. 7A, *gray traces*). When we added ATP to a preformed binary complex of GroEL SR-1 RW and MDH unfolded in Gdn-HCl, we found that the amplitude of each transition was greatly decreased compared to double ring GroEL R231W. The presence of MDH suppressed the amplitude of both Phase B and Phase C, with the amplitude of Phase C decreased to one fifth of that seen in the absence of bound substrate (Fig. 7A, *“-GroES”*, compare *black* and *gray* traces; also see Table S1 for values). In contrast, in the presence of unfolded MDH in amounts sufficient to bind both rings of the GroEL 14-mer, GroEL R231W displayed an analogous suppression in amplitude, but in the case of GroEL this effect was specific to Phases C and D, as noted previously [20]. Another difference between the single ring and double ring forms of GroEL was that this suppression of amplitude was also observed for Phase S, observed when GroES was added to the reaction (Fig. 7A, *“+GroES”*). Double-ring GroEL R231W did not display such amplitude suppression (Fig. 7B, *“+GroES”*).

### Release of Unfolded Proteins from GroEL Mutants

**Figure 8:**
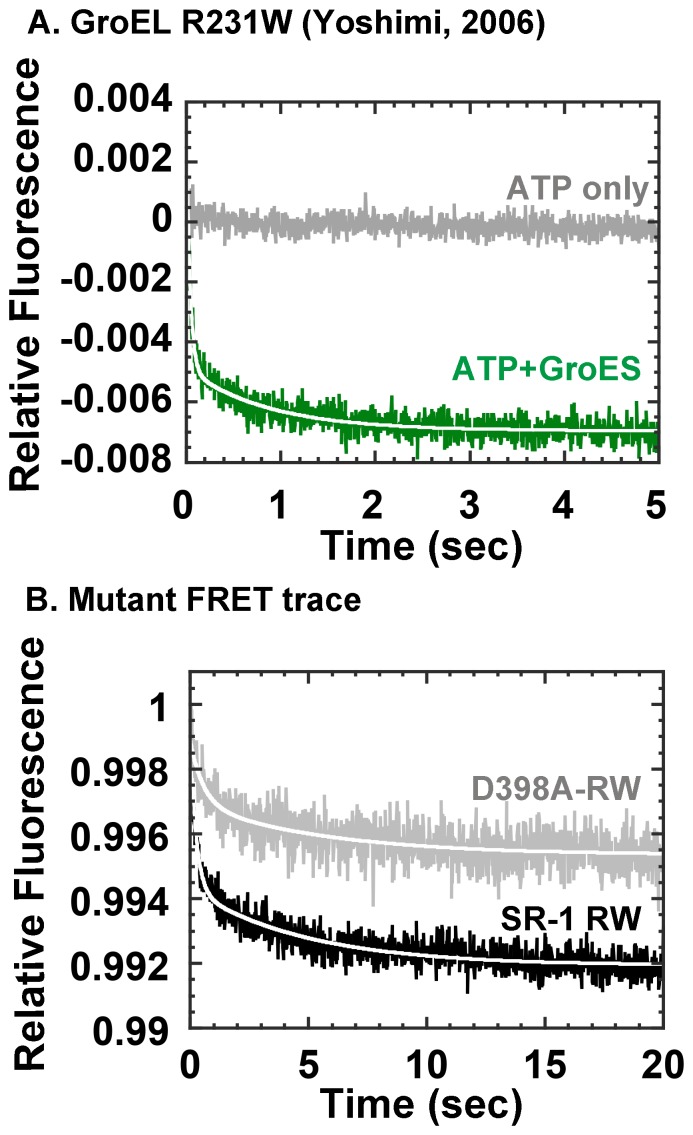
FRET analysis of subunit protein release triggered by ATP and GroES. Fluorescence intensities at wavelengths greater than 420*et al* [20]. *Gray trace* indicates changes in FRET fluorescence in the presence of ATP only (no GroES), *green trace* indicates FRET changes in the presence of ATP and equimolar GroES. B. FRET traces for mutants. *Black trace* indicates SR-1, and *Gray trace* indicates D398A-RW. Note the differences in time scale between panels A and B.

Do the changes in kinetic profiles seen in the D398A-RW and SR-1 RW mutants translate to effects further downstream of the chaperonin mechanism? To address this we used intermolecular FRET to probe the rate at which unfolded protein molecules are detached from the GroEL apical domain of these mutants upon ATP and GroES addition. We used a protocol described in a previous study [20] where we used acid-unfolded wild type GroEL (GroELwt) labeled with IAEDANS as a model substrate protein with fluorescence acceptor, and the tryptophan introduced to the apical domain of each mutant as fluorescent donor (Fig. 8A, *green trace*). We found that the rate of unfolded release was in fact slowed significantly in both D398A-RW (Fig. 8B, *gray traces*) and single-ring SR-1 RW (Fig. 8B, *black trace*), compared to the original double ring chaperonin (Fig. 8A). Compared to GroEL R231W (which displayed a biphasic decrease in FRET with rate constants of *k*_1_ = 27.0 s^−1^ and *k*_2_ = 1.16 s^−1^, respectively, Fig. 8A [20]), upon addition of ATP both D398A-RW and SR-1 RW showed fluorescence decreases that were 4- to 15- fold slower (SR-1 RW, *k*_1_ = 4.1±0.29 s^−1^ and *k*_2_ = 0.21±0.012 s^−1^; D398A-RW, *k*_1_ = 1.8±0.46 s^−1^ and *k*_2_ = 0.18±0.034 s^−1^, respectively, Table 2). The FRET analysis suggested that in both cases, mutation caused a deleterious effect on the smooth release of unfolded protein from the apical domain of the GroEL ring.

## Discussion

In a recent study, Clare and coworkers used cryo-EM to identify numerous structural substates that were formed by GroEL D398A upon ATP binding [13]. Their results showed that after binding of ATP, GroEL forms a number of alternate conformations that involve rearrangements of the apical domain and the formation of alternate salt bridges that lead to the formation of the final open, GroES bound structure determined by X-ray crystallography. The four major asymmetrical configurations of GroEL, T, Rs_1_, Rs_2_, and R-open, represented a sequence of “click-stop” molecular events that lead to the formation of the final encapsulation complex.

In this study we were interested in constructing a kinetic view of the same process immediately after ATP binding to GroEL that would add upon the findings revealed in Clare *et al* [13]. Our chosen method was to probe the dynamic behavior of a series of mutants that have displayed interesting functional deviations from wild type GroEL and, by comparing the kinetic behavior of these mutants in stopped-flow studies, reconstruct a similar molecular trajectory that describes the encapsulation process. Our results are in good agreement with the results of Clare *et al.* and support a sequential mechanism that leads up to the formation of an open GroEL conformation with bound GroES. We also found additional hints indicating that the process of substrate protein encapsulation involves interactions between multiple locations in the GroEL subunit that interlock smoothly to ensure proper function.

### GroES Binding and Protection of Unfolded Substrate may Proceed without Large-scale GroEL Conformational Changes

Circular permutation has been used with great success to probe various structural elements that are essential for correct folding of protein targets [28], [29], and has also proven to be an effective method to introduce novel and useful functional abilities in a number of proteins, a prominent example being the fluorescent GFP protein [30]. A recently isolated circular permutation mutant (CP86) of GroEL displayed curious characteristics that deviated significantly from the behavior of wild type GroEL, so we selected this mutant as our first candidate for analysis. The most interesting functional difference that CP86 displayed was an apparent change in specificity toward refolding proteins; GroEL CP86 could assist the refolding of the stringent substrate MDH to levels indistinguishable from wild type (Fig. 2A), and yet almost completely lost the ability to assist rhodanese refolding (Fig. 2B). This shift in substrate specificity was apparently caused by the disruption of the polypeptide backbone through permutation, as introduction of cysteines to the polypeptide ends and subsequent oxidizing treatment resulted in a recovery of the ability to assist rhodanese (Fig. 2B).

When we probed the molecular basis of this change in substrate specificity, we found that CP86 displayed a very restricted version of the kinetic transitions that were detectable by stopped-flow analysis in wild type (Figs. 4A, B), consisting of an initial rapid fluorescence increase (Phase A) followed by a very slow decrease (Phase B?). Nevertheless, CP86 was capable of binding GroES and sequestering unfolded rhodanese molecules from Proteinase K digestion (Figs. 4C and D). This result provided three additional hints to the molecular puzzle: first, CP86 was capable of binding and protecting rhodanese molecules from aggregation, but was unable to release them for productive refolding, most likely due to the inability to alter its structure for productive release. Second, CP86 was still capable of assisting the productive folding of another stringent protein, MDH, in spite of such restrictions in structural dynamics (Fig. 2A). This result reflects a previously undetected difference in the refolding mechanisms of two stringent refolding proteins, and hint that there may be multiple routes through the chaperonin mechanism that lead to productive folding. And finally, since CP86 was capable of binding GroES and sequestering unfolded rhodanese from Proteinase K in spite of the near complete lack of detectable conformational changes, there is a distinct possibility that GroES binding and subunit sequestration precedes dynamic rearrangements of the GroEL apical domain in the native chaperonin mechanism as well.

This last assertion must be qualified to avoid mistaken interpretations. A simple explanation of the results seen in Fig. 4 may be that as a result of circular permutation, the configuration of the apical domain ring may have changed relative to the original wild type configuration, and as a consequence, GroES binding may proceed without a specific conformational transition such as the Rs_1_ to Rs-open conversion proposed by Clare *et al* [13]. This possibility needs to be clarified by performing actual detailed structural studies on CP86, perhaps by using the cryo-EM methods used by these authors. We note, however, that the original CP86 mutant displayed structural characteristics that were similar to wild type GroEL in many experiments that we performed, for example, denaturant stability (Fig. 3B), and resistance to Proteinase K digestion (Fig. 4C). GroEL CP86 is also largely unable to bind GroES in the absence of ATP (confirmed during purification), unlike other mutations that we have characterized such as GroEL G192W [31]. The fact that we could engineer a disulfide bond into CP86 so that it mimics the wild type chaperonin seems in itself evidence that steric perturbation caused by this mutation was kept to a minimum, and it is worthwhile to consider the possibility of an early-stage GroES binding event.

Another interesting, highly speculative possibility that we wish to put forth related to the above discussion is that the two events, GroES binding/protein sequestration and GroEL subunit rearrangement, are not arranged in a sequential manner in the mechanism but instead proceed *in parallel* and are initiated by a common trigger, the binding of ATP. In such a case, in CP86 the mutation may have caused the loss of only one of these parallel events while retaining the other. We see some evidence to support this idea from the behavior of the GroES-dependent Phase S throughout our experiments. As seen in Figs. 4B (for CP86) and 7A (for SR-1), as well as in experiments of a previous study using CP376 [22], the detection of Phase S is reproducible in a variety of mutants with altered structural characteristics, and this suggests that binding of GroES proceeds regardless of various limitations imposed on GroEL through mutation. This behavior is consistent with a co-chaperonin binding event that is largely independent of the kinetic transitions detected in the latter portion of our experiments. An interesting experiment would be to perform cryo-EM observations on GroEL in the presence of GroES, to see if multiple substates similar to those elucidated by Clare *et al.* may also be detected when GroES is bound to GroEL.

In wild type GroEL, Gly86 is located in the conserved phosphate-binding loop, which is an essential structural element in the ATP hydrolysis mechanism of GroEL. This region has been highlighted in a previous stopped-flow study performed by Kovács and coworkers on a single-ring variant of GroEL [32], and we observed a number of common characteristics between their results and ours which merit comparison. The SR-A92T mutant characterized by Kovács *et al*. displayed a greatly restricted kinetic profile compared to wild type when probed using a tryptophan residue situated in the base of the equatorial domain (at position 485). Their characterization of SR-A92T was very similar to our evaluation of the kinetic behavior of GroEL CP86; an initial very rapid transition at the upper boundaries of stopped-flow analysis was retained, and a single kinetic phase that was greatly attenuated relative to the wild type was observed, as was a loss of all subsequent phases. Although we should be careful of comparing experimental results of different mutants using fluorescent probes located a different positions of GroEL, we believe that the similarities in kinetic characteristics between GroEL SR-A92T and the circular permutant CP86 are not a coincidence. Perhaps the phosphate loop and adjacent regions represent a point where signals of ATP binding and hydrolysis propagate outward toward the intermediate and apical domains to initiate domain rearrangements. Disruption of this region, either through point mutations or circular permutation, would result in altering a significant portion of this intra-subunit communication network, with consequences to the overall dynamics and functional characteristics of GroEL. We observe with interest that, apart from the details seen in stopped-flow studies, many of the functional aspects of GroEL differ between GroEL SR-A92T and CP86 (for example, binding of GroES stimulates the ATPase activity of GroEL SR-A92T; the same event suppresses the ATPase of CP86, Fig. 2C), hinting at the potential complexity of the overall mechanism. Nevertheless, the relationship between the GroEL phosphate loop region and the dynamic behavior of the GroEL subunit is an interesting subject for further experiments.

### Apical Domain Tilting and Orientation of Helix M in GroEL

GroEL D398A has been used extensively in experiments to probe GroE function, owing to the fact that the rate of ATPase activity in this mutant is greatly reduced compared to wild type [10], [11]. This allows researchers to isolate and characterize GroEL molecules in various intermediate states of the mechanism. In an attempt to utilize these characteristics in stopped-flow studies, we next turned to the characterization and analysis of GroEL D398A-RW.

Our experiments showed that the effects of the D398A mutation were reflected particularly strongly in a specific kinetic transition of the apical domain, Phase C (Fig. 5A). This phase has been shown in various previous experiments to display numerous unique characteristics. For example, in Yoshimi *et al.* we found that the rate of Phase C decreases in the presence of prebound unfolded MDH molecules, which indicated that this phase is sensitive to the steric load of proteins bound to the apical site ([20], see also Fig. 7B, “*-GroES*”). Also, in GroEL CP376, we found that shifting the polypeptide ends of GroEL to the hinge that connects the apical and intermediate domains resulted in a complete and highly specific elimination of this phase from the kinetic profile [22]. These results are all consistent with the idea that Phase C represents a large tilting movement centered in the apical domain that presumably leads to the open configuration characterized in the X-ray crystal structure of the GroEL-GroES-ADP_7_ complex [5].

Using GroEL D398A-RW, we now show that this transition may be closely correlated with the rearrangement of Helix M in the intermediate domain (colored in *orange* in the subunit model shown in Fig. 1, *center right*). As shown in Fig. 5B, we found that in GroEL D398A-RW, the rate of Phase C was decreased more than one-hundredfold, to *k = *0.019±0.00029 s^−1^, compared to *k* = 2.1±0.11 s^−1^ for GroEL R231W. This drastic decrease in apparent rate immediately suggested to us a possible correlation with the strong decrease in steady-state ATP hydrolysis displayed by this mutant. However, the rate of this phase was about tenfold higher than the most conservative estimates of the steady-state ATPase rate of GroEL D398A (based on an apparent rate of *k* = 0.12 s^−1^ [33], a 50-fold decrease in ATPase rate would correspond to an apparent steady state ATPase rate of *k* = 0.0024 s^−1^), which would argue against the notion that Phase C represented the rate-limiting step of ATP hydrolysis. To clarify this point, we performed stopped-flow experiments by substituting AMP-PNP for ATP, and found that Phase C became undetectable in the presence of this analog (Fig. 5B).

At first glance, this result seemed to support a mechanistic dependence on the part of Phase C toward ATP hydrolysis. However, since such a dependence should also apply to the wild type chaperonin, we tried to confirm this relationship using GroEL R231W and found that, although AMP-PNP caused a general reduction in the apparent rate constants of all of the observable phases (Fig. 6A and B), none of the phases were selectively affected as seen for Phase C in GroEL D398A-RW. The substitution of AMP-PNP for ATP seems to cause a general decrease in apparent rates throughout the mechanism, which might be explained for example by an incomplete binding of this analog to the GroEL ATP binding site.

In their characterization of the numerous substates formed after ATP binding to GroEL, Clare *et al.* noted that a significant molecular event in the T to Rs_1_ transition was the reorientation of Helix M, containing Asp398 within its structure, to a position close to the ATP binding site. The side chain of Asp398 would interact with the gamma phosphate group of ATP, and this interaction would act to stabilize the orientation of Helix M in the latter configuration. If we postulate that this reorientation of Helix M acts as a “speed boost” mechanism for Phase C, it would nicely explain the selective retardation of this phase shown in Fig. 5. This mechanism is not strictly necessary for completion of the chaperonin mechanism, since D398A is able to complete all of the apparent molecular steps leading to encapsulation, albeit very slowly. Probable additional factors that would propel the chaperonin cycle along this trajectory would be multiple binding events between the GroEL apical domains and GroES, and the rearrangement of various intersubunit salt bridges. The orientation of Helix M to the GroEL ATP binding site seems to be important specifically for the rapid rearrangement of the GroEL apical domain represented by Phase C, which in turn is implicated via previous experiments to be involved in the formation of the final encapsulating form (R-ES in Clare *et al*. [13]).

This relationship between Phase C and Asp398 may act as an important control mechanism that ensures efficient folding assistance. Previous studies have indicated that under normal conditions in the cell, ATP would be likely to bind to GroEL first, rather than unfolded polypeptide [34]. A multi-stage mechanism that links apical domain movement with molecular rearrangements that follow ATP binding, instead of a simple mechanism that triggers immediately upon ATP binding, would serve to increase the time that ATP-bound forms of GroEL could acquire unfolded protein in a productive state. The “speed boost” idea is also consistent with the idea that the conformational substates detected in GroEL D398A using cryo-EM precede the “power stroke” transition that lifts the apical domain into the open conformation [13].

The rearrangement of Helix M that is facilitated by Asp 398 may be important to an additional facet of the GroEL machinery that has been highlighted in two very recent studies of GroEL. Fei and coworkers have shown that a mutant GroEL (GroEL^D83A/R197A^) that lacks the ability to form a specific inter-subunit salt bridge (R197-E386) displays a markedly asymmetric distribution of apical domain configurations about the GroEL ring when ADP is bound [35]. The functional implications of such asymmetry was highlighted in experiments performed by Chen *et al.,* who showed in cryo-EM experiments that individual apical domains of a GroEL ring may perform different functions such as GroES binding and unfolded protein recognition, thereby breaking the seven-fold symmetry implied in the basal GroEL structure [36]. These two studies point toward the interesting notion that the arrangement of apical domains about the GroEL heptameric ring may be intrinsically asymmetric in nature. The R197-E386 salt bridge is disrupted by rearrangement of Helix M during the T to Rs_1_ transition, and so the results of Fei *et al.* suggest that this transition might be conductive to a more dynamic, asymmetric arrangement of apical domains about the heptameric ring, which would facilitate the functional heterogeneity of apical domains observed by Chen and coworkers. Phase C, in this context, may be reflective of the relative ease by which such asymmetry is achieved after ATP binding.

### The Double-ring Structure of GroEL Aids Efficient Protein Release from the Apical Domain

The third mutant used in this study (GroEL SR-1 RW) was initially characterized along with GroEL R231W in Taniguchi *et al.* [21]. Our new experiments indicated that in SR-1, the presence of bound protein profoundly affected the dynamic behavior of the apical domain. This effect of bound protein is seen in R231W as well, but only for Phase C. In the case of SR-1, both Phases B and C were affected, and the changes could be characterized as large decreases in amplitude, rather than rate. Also, in the case of GroEL R231W, GroES binding could offset the decrease in rate of Phase C brought about by unfolded peptide binding. However, in SR-1 RW a ∼50% decrease in amplitude was detected for the GroES-dependent Phase S as well (Fig. 7A, *right*), indicating that GroES binding was also affected by the absence of the second chaperonin ring in this mutant. We conclude from this that the integrity of apical domain movement in GroEL relies significantly on the presence of both rings of the GroEL oligomer. A very naïve mechanistic interpretation of this result would be that the rings of GroEL interact with each other to form a “base” by which GroEL can exert force on the unfolded protein via apical domain movements. In SR-1, this base is absent, and the consequence of this would be an inability to exert sufficient force and complete the transition smoothly. Although naïve, this explanation fits nicely with an important functional hypothesis of the chaperonin molecular mechanism, that of forced unfolding of bound substrate proteins by apical domain rearrangement [37], [38]. Application of mechanical force to molecules may well require the presence of a stable “base” to allow transfer of force to proteins that result in a net unfolding effect. We note with interest that Clare *et al.* viewed an expansion and rotation of the GroEL heptameric ring in the structural transitions of GroEL D398A triggered by ATP binding, that are centered around the equatorial domain interface [13] which would of course be missing in SR-1.

In light of our results regarding the effects of unfolded protein on the apical domain movements of SR-1, we note with great interest that active variants of single ring GroEL have been derived through additional mutations to SR-1 that decrease the affinity of GroES toward GroEL [32], [39], [40]. This indicates that the arrest of the chaperonin cycle observed in SR-1 might be attributable to a GroEL-GroES interaction that is too strong to be overcome by the single ring form. This would agree very well with our present idea that single ring forms of GroEL are inherently sensitive to protein loads and are very slow to, and in some cases incapable of, dislodging bound molecules (such as unfolded polypeptide or GroES) from the apical domain.

### Impaired Subunit Dynamics Affect Substrate Protein Release from the Apical Domain

Using FRET between bound substrate protein and the apical domain tryptophan, we found that in D398A and SR-1, the release of bound proteins from the initial binding site were hampered as a consequence of mutation. In each case the effects brought about were similar; detachment of proteins from the apical domain were slowed by a factor of 7 to 15 compared to R231W. In the present experiments, we chose acid-denatured wild-type GroEL as an unfolded protein substrate, and the relatively large size of the substrate in this case allowed the detection of a clear FRET signal. At the same time, this large size likely precludes encapsulation of substrate protein after release, so our present results should be interpreted to reflect early release events that take place as a consequence of GroEL domain conformational change.

It was interesting that decreased ATPase activity (D398A) and single-ring conversion (SR-1) both resulted in a decrease in the rate of protein release from the GroEL apical domain ring. For D398A, it is likely that the greatly decreased rate of Phase C is contributing to a decrease in the rate of protein release, a downstream event whose completion is likely required for the formation of the open encapsulation complex. In the case of SR-1, the increased sensitivity of GroEL domain movement toward bound protein is likely restricting the completion of this same complex, resulting in a similar decrease in release rate. These two disparate factors, one structural, one functional, act on the mechanism to ensure the rapid and smooth release of polypeptides from the apical domain ring that is so crucial to the successful completion of the chaperonin mechanism.

## Conclusions

**Figure 9:**
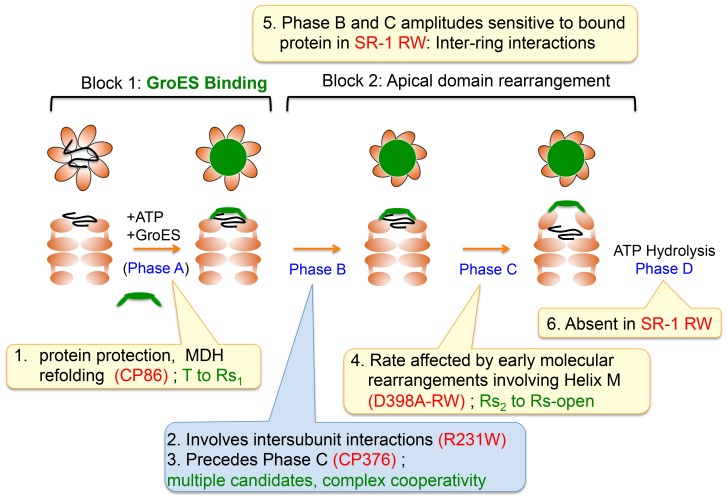
Proposed sequence of events for GroEL encapsulation, based upon results from the present study. In this figure, ideas derived from the present study are illustrated in *yellow text bubbles*, ideas based upon prior studies [20]–[22] are illustrated in the *blue text bubble*. The scheme is based on the characteristics displayed by numerous mutant forms of GroEL that highlight certain dependencies between individual molecular events. Kinetic phases detected in our stopped-flow experiments are indicated in *blue text*, names of individual mutants are denoted in *red text*, and *green text* indicates proposed correlations and conclusions derived from comparisons of our results with various previous studies. Details are given in the main text.

Summarizing our results, we propose a plausible sequence of events that follow ATP binding to GroEL in Fig. 9. The basis for our proposed sequence lies in our characterization of a series of GroEL mutants that have lost specific portions of this sequence while retaining others, which highlighted dependencies among molecular events. Our results from the circularly permuted mutant CP86 suggested that the GroEL mechanism may be separated into two event blocks, with GroES binding and protection of the unfolded protein occurring first, followed by large scale rearrangement of the GroEL apical domain and subunit orientation. Regarding this second block, we have shown in a previous study using GroEL CP376 that Phase B most likely precedes Phase C in the mechanism [22]. The cooperative nature of this phase suggests that it involves intersubunit interactions such as the rearrangement of salt bridges seen by cryo-EM. With regard to Phase C, our results from D398A-RW in the present study suggest that the rate of this phase may be strongly dependent on the orientation of Helix M to the ATP binding site. Clare and coworkers propose that this rearrangement occurs very early in the chaperonin cycle. Phase D, the slowest kinetic transition that was detected in our experimental system, most likely follows after this second event block is completed, along with the hydrolysis of ATP. In our most recent experiments we have seen that Phase D is observed only in double ringed forms of GroEL (Fig. 7A and B, compare the *black traces* in the *left panels*), and so it may be that this phase is involved in inter-ring communication, possibly leading to a second cycle on the opposite ring of GroEL.

It is difficult to correlate the results of our experiments completely with the detailed structural aspects of the GroEL substates elucidated by Clare and coworkers [13]; nevertheless we believe that some inferences may be made (Fig. 9, highlighted in *green text*). Our results from CP86 indicate that GroES binding to GroEL may be possible at a very early stage of the cycle. The initial T to Rs_1_ transition seen by Clare *et al.* is the earliest structural event of the cycle detected in cryo-EM experiments, and from this we believe that this transition may represent the conformational switch that allows GroES binding. In this case, from stopped-flow experiments on CP86 in Fig. 4A, we see that only Phase A, the initial rapid fluorescence increase that occurs in the dead zone of our 5-sec assay, remains the single kinetic transition that is detectable in this mutant, and so this phase might represent the T to Rs_1_ transition. Correlating Phase B with a specific structural transition proves to be more difficult, since Clare and coworkers have documented multiple structural transitions that involve the rearrangement of salt bridges between adjacent subunits (the T to Rs_1_ transition and the Rs_2_ to Rs-open transition) as well as a complex rearrangement of salt bridges between the two GroEL rings [13]. Any of these rearrangements may conceivably correspond to Phase B, and at present we lack the necessary details to attribute this kinetic phase to a specific structural event. The characteristics of Phase C suggest that this kinetic transition involves a very large rearrangement of the apical domain that would be conceptually sensitive to the presence of bound unfolded protein, and this characteristic, compounded with the rate of this phase compared to the other detected kinetic transitions, would seem to suggest a relationship between Phase C and the Rs_2_ to Rs-open transition. Our results from the D398A-RW mutant suggest that the rate of this transition differs depending on the stability or the specific orientation of Helix M in the intermediate domain.

Our proposed mechanism does not have a clear-cut correlation with the structural data that has been reported to date; specific aspects of the mechanism remain mismatched or missing. Also, our proposal disagrees with the mechanism proposed by Clare *et al.* in one important aspect, the specific instance that GroES binds to the GroEL ring. We believe that our data indicates that GroES binding and subsequent protection of the unfolded protein may be accomplished surprisingly early in the mechanism. Such a mechanism would provide advantages to the refolding protein molecule, for example, improved chances of being successfully incorporated into the chaperonin cavity, and the beneficial effects of increased interactions between substrate and chaperonin that may affect the refolding trajectory of the bound protein. The merits of both of these concepts have been discussed in various previous studies, and hopefully our result will aid in clarifying their significance in the overall mechanism.

## Supporting Information

Table S1 (XLSX)
